# Deep learning for multitask prediction on thyroid nodule frozen sections

**DOI:** 10.3389/fonc.2025.1676360

**Published:** 2026-01-12

**Authors:** Chunyang Wang, Juan Hu, Xiang Li, Yufeng Cai, Shixiang Wang, Xusheng Wu, Haixia Liu, Zhongliang Hu, Dehua Hu

**Affiliations:** 1 School of Life Sciences, Central South University, Changsha, China; 2 Eight-Year Medical Program, Xiangya School of Medicine, Central South University, Changsha, China; 3 The First People’s Hospital of Xiangtan City, Xiangtan, Hunan, China; 4 Shenzhen Health Development Research and Data Management Center, Shenzhen, China; 5 Department of Pathology, Xiangya Hospital, Central South University, Changsha, China; 6 Department of Pathology, Xiangya School of Basic Medical Sciences, Central South University, Changsha, China

**Keywords:** frozen sections, pathological image, thyroid cancer, artificial intelligence, deep learning

## Abstract

**Background:**

Preoperative ambiguous thyroid nodules often depend on intraoperative frozen sections for surgical planning, but misdiagnosis can occur due to low-quality frozen sections, limited diagnostic time, and a shortage of pathologists. Deep learning models and conventional radiomics have shown potential in improving diagnostic accuracy in thyroid nodules, yet their integration remains under-explored. This study aimed to develop deep-learning-based models to assist in the intraoperative pathological diagnosis of thyroid nodules by classifying benign/malignant cases, predicting BRAF^V600E^ gene mutation, and identifying lymph node metastasis.

**Methods:**

A total of 436 Whole-Slide Images (WSIs) of thyroid frozen sections were analyzed using deep learning techniques. The analysis included image preprocessing, feature extraction, and classifier training. Patch-to-WSI feature aggregation was done via Patch Likelihood Histogram (PLH) and Bag of Words (BoW) methods.

**Results:**

On the test set, the InceptionV3 model performed best in benign/malignant classification with an AUC of 0.998 and accuracy of 0.988, where weakly supervised strategies surpassed supervised ones. For BRAF^V600E^ gene mutation prediction, the ResNet50 model achieved a patch-level AUC of 0.831 and a WSI-level accuracy of 94.4% under the extended strategy. A ViT-based model for lymph node metastasis prediction obtained an AUC of 0.671 and accuracy of 76%.

**Conclusions:**

The study indicates that deep learning models can effectively classify benign/malignant thyroid frozen sections, predict BRAF^V600E^ gene mutations, and predict lymph node metastasis status. It also emphasizes the effectiveness of weakly supervised strategies in thyroid lesion frozen sections, which could lessen reliance on pathologists’ annotations.

## 1 Introduction

The overall prevalence of thyroid nodules in the general population is 24.8% (1), of which thyroid cancer is the most common malignant lesion of thyroid nodules (2), often presenting symptoms such as dysphagia, dyspnea, and local pain, and is prone to distant metastasis in later stages, severely affecting patients’ quality of life. The incidence rate of thyroid cancer exhibits a complex global trend, with increases observed in some regions and declines in others (3, 4). The incidence rate of thyroid cancer is generally higher in females than in males (5), and significant differences exist across countries and regions (5, 6).

With the continuous development of scanning, digital, and computer technologies, the emergence and application of high-quality digital pathological sections images, especially Whole-Slide Image (WSI) data, have greatly promoted the development of the pathology field, and related research on digital pathological images has garnered widespread attention (7). The proposal and development of artificial intelligence (AI) have made it possible for AI to assist in pathological diagnosis. Deep learning (DL)-related methods have been widely applied in the analysis of pathological images, such as classifying, segmenting, and detecting basic elements (e.g., cells, glands), and identifying morphology-related prognostic features (8), providing assistance for objective diagnosis and prognosis prediction in digital pathological images. However, most AI pathological studies are based on paraffin sections, and there is a significant gap in research on frozen sections. Among specific tumor types, the breast cancer (9, 10) field is one of the earliest to develop AI pathology, while other more studied tumors include lung cancer (11, 12), prostate cancer (13, 14), gastrointestinal tumors (15, 16), and neurological tumors (17, 18), with relatively few studies on thyroid cancer.

The most common application of AI-assisted diagnosis is in determining the benign/malignant tumors, which is a fundamental requirement for AI-assisted pathology. AI-assisted diagnosis related to thyroid cancer also includes tasks such as predicting BRAF^V600E^ gene mutation and predicting lymph node metastasis. The BRAF gene, formally known as V-Raf murine sarcoma viral oncogene homolog B, promotes cell proliferation, differentiation, and apoptosis and was first described in 1983 (19). The V600E mutation is the most common type of BRAF mutation and is the most frequently occurring mutation in thyroid cancer patients. Studies have demonstrated that the BRAF^V600E^ mutation plays a diagnostic role in improving the risk assessment of malignancy and treatment options for patients with “indeterminate” cytological thyroid nodules (20). Lymph node metastasis (LNM) is a common clinical manifestation of thyroid cancer and is also an important indicator for evaluating patient prognosis and treatment plans. Preoperative detection of LNM in thyroid cancer primarily relies on neck ultrasound and neck computed tomography (CT) scans. However, the accuracy of ultrasound and neck CT in detecting cervical LNM is limited, with 60-70% of central LNM being missed by neck ultrasound or CT (21).

Therefore, we analyzed thyroid frozen sections WSI using deep learning methods to explore intraoperative pathological auxiliary diagnosis. Our goal was to preliminarily determine tumor benignity/malignancy, BRAF^V600E^ gene mutation status, and LNM to assist intraoperative pathological diagnosis effectively.

## 2 Materials and methods

### 2.1 Dataset

We used WSI images of thyroid frozen sections from Xiangya hospital affiliated with Central South University. Intraoperative thyroid lesion tissues were collected from patients and processed into frozen sections through a series of procedures including freezing, sectioning, and staining. After preparation, the pathological sections were scanned using a Pannoramic Scan scanner from 3D Histech (Hungary) at 40× magnification, corresponding to a pixel size of 0.25 μm × 0.25 μm. The resulting digital pathology images were stored in mrxs format.

In this study, sample labeling was based on postoperative paraffin histopathological diagnosis, while the goal of model training was to serve intraoperative diagnosis. Slides diagnosed as normal thyroid tissue, nodular hyperplasia, nodular goiter, subacute thyroiditis, Hashimoto’s thyroiditis, or thyroid adenoma were labeled as benign, while those diagnosed as papillary thyroid carcinoma, medullary carcinoma, poorly differentiated thyroid carcinoma, etc., were labeled as malignant. We present the clinical diagnosis and benign/malignant classification for all samples (**Supplementary Table S1**). Follicular neoplasms were not included in the study because they cannot be definitively diagnosed by intraoperative frozen section. WSI images were annotated by pathologists with extensive experience using QuPath software to mark Regions of Interest (ROI), and reviewed by another pathologist.

The dataset included 335 malignant samples and 101 benign samples with ROI regions. Some WSI images also contained BRAF^V600E^ gene mutation detection results (301 mutated samples, 29 non-mutated samples) and information on lymph node metastasis (185 metastatic samples, 147 non-metastatic samples). Despite the limited sample size, the deep learning in this study is performed at the patch level. A single WSI image can be segmented into thousands of patches, which satisfies the requirements for patch-level deep learning. Therefore, these WSI images and annotation information were used to construct models for the preliminary classification of thyroid sections benignity/malignancy, prediction of BRAF^V600E^ gene mutation in thyroid cancer, and prediction of lymph node metastasis.

### 2.2 Image preprocessing

Single WSI image can have a resolution of around hundreds of billions of pixels, making it difficult to directly input complete WSI images into deep learning models for training. Therefore, it is necessary to crop WSI images into patches for deep learning model training. Using a sliding window method at a 20× magnification, the original images were sequentially cropped into 512×512 resolution patch images, which contained ratio information of the ROI regions. To filter tissue patches, we set color thresholds to remove blank patches and used a trained ResNet50 model to distinguish between tissue and non-tissue patches. Meanwhile, to reduce color differences in WSI images caused by instruments and operators, the Vahadane method was selected for color standardization.

In the classification of thyroid benignity/malignancy, patch labels were calculated using both supervised and weakly supervised strategies. Under the supervised strategy, if a patch had over 80% of the ROI annotation area, it was labeled as malignant (label 1); patches not exceeding 80% of the ROI annotation area were discarded, and the remaining patches not including the ROI annotation area were labeled as benign (label 0). Under the weakly supervised strategy, patch labels were consistent with the WSI labels without using ROI annotation information.

In BRAF^V600E^ gene mutation classification, convolutional neural network (CNN) models were trained using both conventional and extended strategies. Under the conventional strategy, only patches from the delineated ROI areas were used as input. In the extended strategy, all patches cropped from the entire WSI were input into the deep learning model for training, and the features learned by the best-performing deep learning model were extracted for WSI-level BRAF^V600E^ gene mutation prediction.

In the classification of lymph node metastasis, all patches extracted from the entire WSI were used as input. Each patch was labeled as metastatic or non-metastatic based on its source WSI and preserved its affiliation with the WSI.

### 2.3 Patch training

In the classification of thyroid benignity/malignancy and BRAF^V600E^ gene mutation, three CNN models—ResNet50, InceptionV3, and VGG16—were selected as patch-level classifiers. The number of fully connected layer nodes before the Softmax activation layer of these three CNNs was modified to 2 according to the requirements of the patch classification tasks. Additionally, transfer learning based on ImageNet dataset pre-trained weights was used to initialize model parameters. The experimental environment for this study consisted of an AMD Ryzen 7 3700X 8-Core Processor (3.59 GHz), 32.0 GB RAM, and an NVIDIA GeForce RTX 2080 Ti GPU with 11.0 GB VRAM. For the same classification tasks, the same parameter settings were applied. The optimizer used was Stochastic Gradient Descent, the loss function was Cross Entropy, the learning rate of the three deep CNNs was set to 0.001, the batch size was set to 32, and all models were trained for 20 epochs.

In the classification of lymph node metastasis, two methods were used. First, InceptionV3 was selected as the patch-level classifier and transfer learning based on the ImageNet dataset was applied. Second, the Vision Transformer (ViT) network was used as a feature extractor to better represent global dependencies in pathological images. ViT extracted 768-dimensional feature representations for each patch, and all patches of the WSI were used as input to predict the category of all WSIs in a weakly supervised strategy.

### 2.4 Feature fusion and machine learning

The Patch Likelihood Histogram (PLH) pipeline and Bag of Words (BoW) pipeline were used to aggregate features from multiple patches to a single WSI. In PLH, the occurrence probability histogram of patches was used to represent the WSI, effectively capturing the distribution of likelihoods through discretization of likelihoods and serving as a representative of the WSI. The BoW pipeline was based on vocabulary technology, where each patch was mapped to a Term Frequency–Inverse Document Frequency (TF-IDF) floating-point variable, and the TF-IDF feature vector was calculated to represent the WSI. Subsequently, the features obtained from the two methods were fused, successfully merging the initially patch prediction results to generate WSI-level features.

The number of features output from the CNN model was excessive, so features with high contribution weights to the final WSI-level prediction were selected. First, features were normalized, and the Pearson correlation coefficient was used to statistically analyze the correlation between features, removing features with a correlation coefficient ≥0.9. Then, Least Absolute Shrinkage and Selection Operator (LASSO) regression was used for feature dimensionality reduction, selecting important features with non-zero coefficients under the optimal parameters.

The features screened in the previous step were used as input features for three models: Logistic Regression (LR), Support Vector Machine (SVM), and Random Forest (RF). Parameter search was used to obtain the best parameters, and the machine learning models were used to predict the test set WSI images.

## 3 Results

### 3.1 Study framework

**Figure 1:**
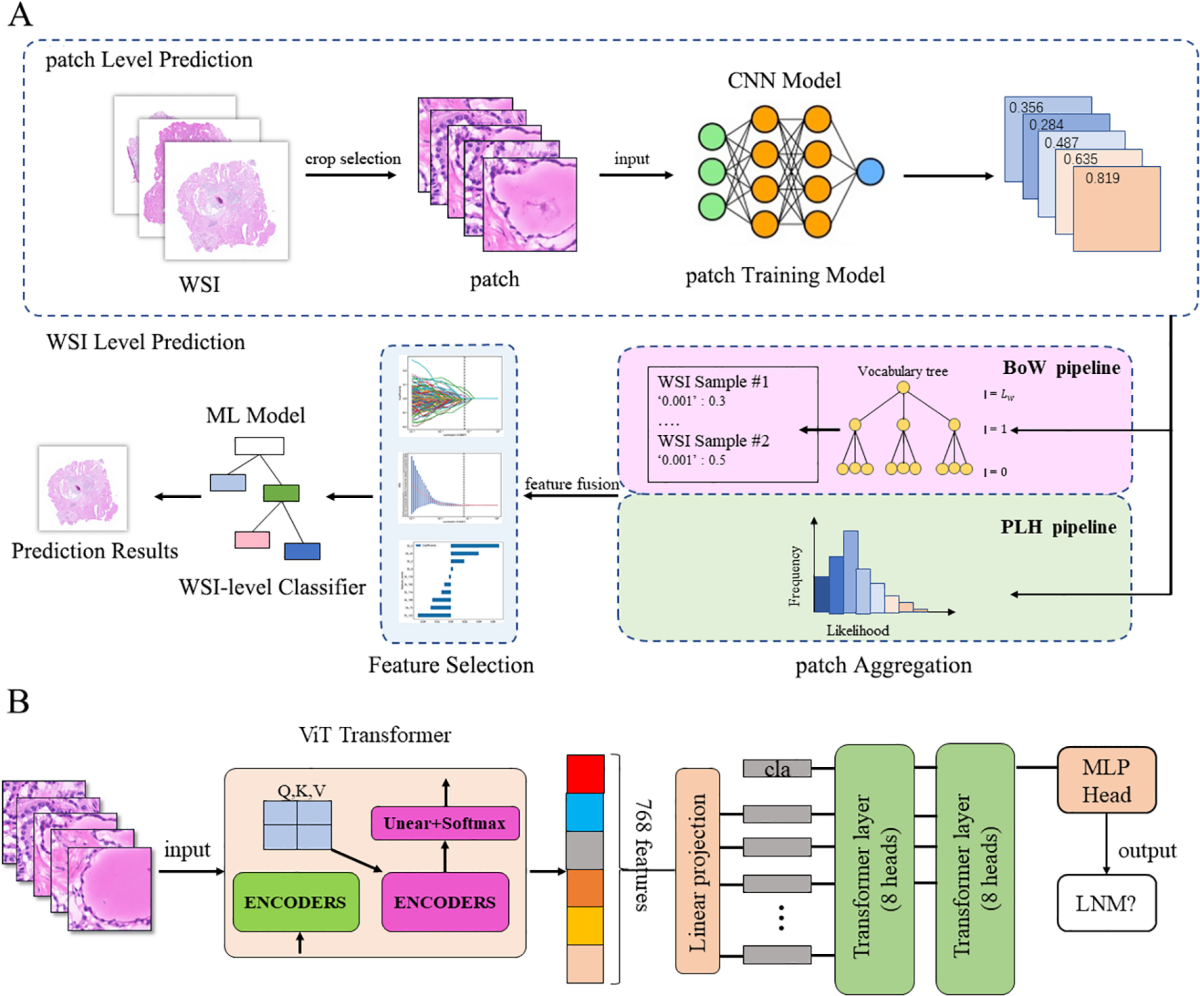
Schematic of the study framework. **(A)** Prediction framework based on CNN-machine learning models. **(B)** Prediction framework based on ViT models. CNN, Convolutional Neural Network; Bow, Bag of Words; PLH, Patch Likelihood Histogram; ML, Machine learning; ViT, Vision Transformer; LNM, Lymph Node Metastasis.

In constructing predictive models for tumor benignity/malignancy, BRAF^V600E^ gene mutation, and lymph node metastasis, we employed a patch-level to WSI-level research pipeline (**Figure 1A**). First, entire WSI images were divided into patches of 512×512 pixels, and each patch was processed through CNN models to generate predictions. Subsequently, patch-level predictions were aggregated into features using the BoW and PLH pipelines. Highly correlated features were selected through LASSO regression. Finally, multiple machine learning models were trained using the selected features as input to perform WSI-level predictions. However, this framework underperformed in predicting lymph node metastasis due to insufficient capture of multiple global features. To address this limitation, we adopted a ViT network as the feature extractor, inputting all patches of the WSI and predicting the class of the entire WSI in a weakly supervised manner (**Figure 1B**).

### 3.2 Benign/malignant prediction of thyroid WSI

**Figure 2:**
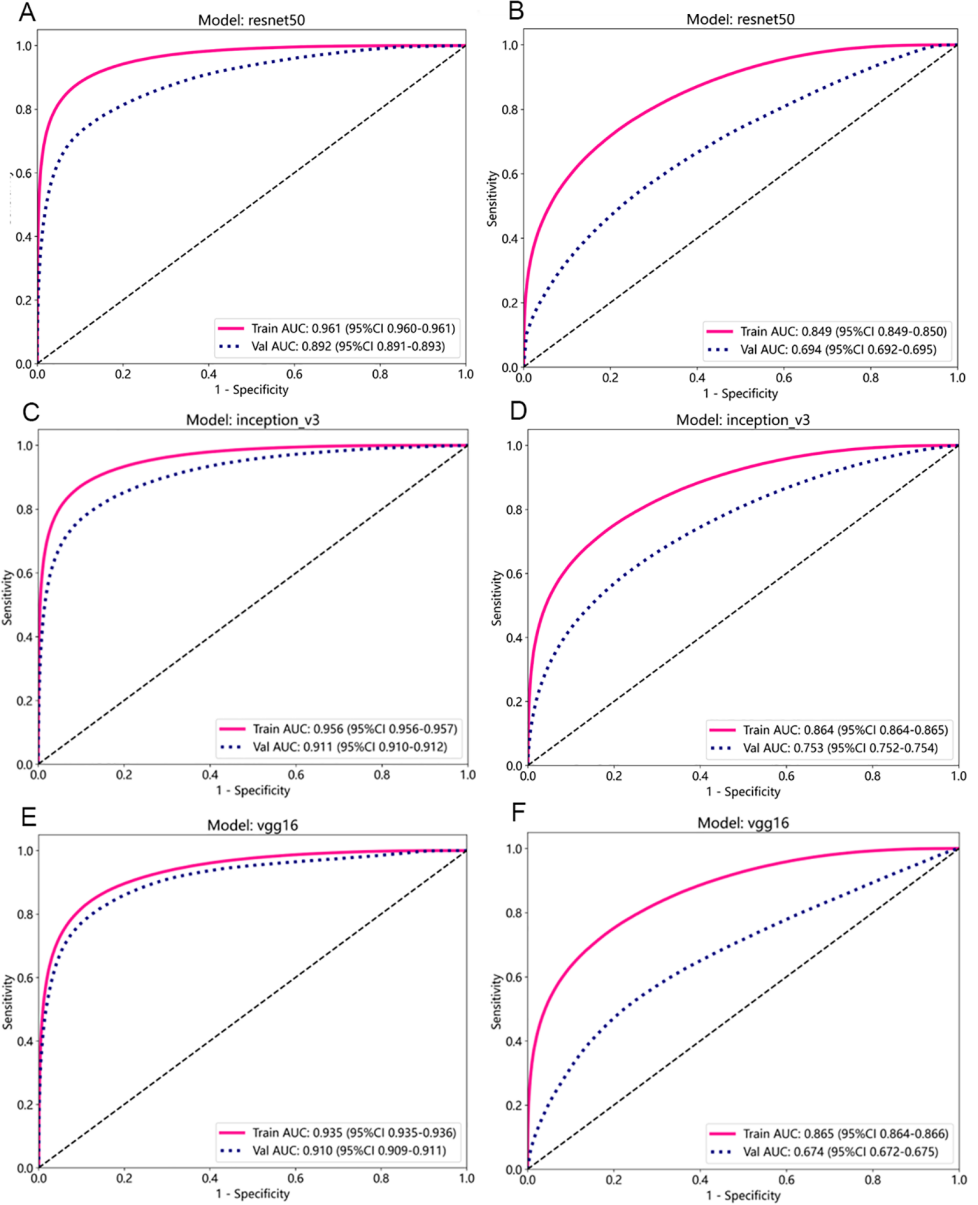
ROC Curves of Different Models in Benign/Malignant Classification. **(A, B)** ROC curves for the ResNet50 model under supervised **(A)** and weakly supervised **(B)** strategies. **(C, D)** ROC curves for the InceptionV3 model under supervised **(C)** and weakly supervised **(D)** strategies. **(E, F)** ROC curves for the VGG16 model under supervised **(E)** and weakly supervised **(F)** strategies.

The performance metrics of the three patch training models under supervised and weakly supervised strategies were compared (**Table 1**), and ROC curves for different models under the two supervision modes were plotted (**Figure 2**). Overall, the three patch training models under the supervised strategy achieved good results, with AUC values on the test set reaching around 0.9. In contrast, under the same training parameters, the three deep learning models under the weakly supervised strategy had AUC values of around 0.7 on the test set, with other metric values also significantly lower than those under the supervised strategy. This may be due to the fact that in the weakly supervised strategy, all patches correspond to the WSI label, leading to a large amount of label noise that affects model performance. Considering AUC values, accuracy, and F1 scores, the InceptionV3 model under the supervised strategy had the highest AUC value, accuracy, and F1 score among the three CNN models, performing the best; under the weakly supervised strategy, the InceptionV3 model had higher AUC and accuracy values than the other two CNN models, with an F1 score slightly lower than the ResNet50 model, making it the best performer. Therefore, the article used the features extracted from the trained supervised and weakly supervised InceptionV3 models.

**Table 1:** Performance of different models in benign/malignant classification.

Training Strategy	Model	AUC	Accuracy	Precision	Sensitivity	F1-score
supervised strategy	ResNet50	0.892	0.853	0.915	0.889	0.902
	InceptionV3	0.911	0.862	0.928	0.887	0.907
	VGG16	0.910	0.856	0.933	0.873	0.902
weakly supervised strategy	ResNet50	0.694	0.601	0.494	0.784	0.606
	InceptionV3	0.753	0.681	0.489	0.782	0.602
	VGG16	0.674	0.653	0.481	0.758	0.589

**Figure 3:**
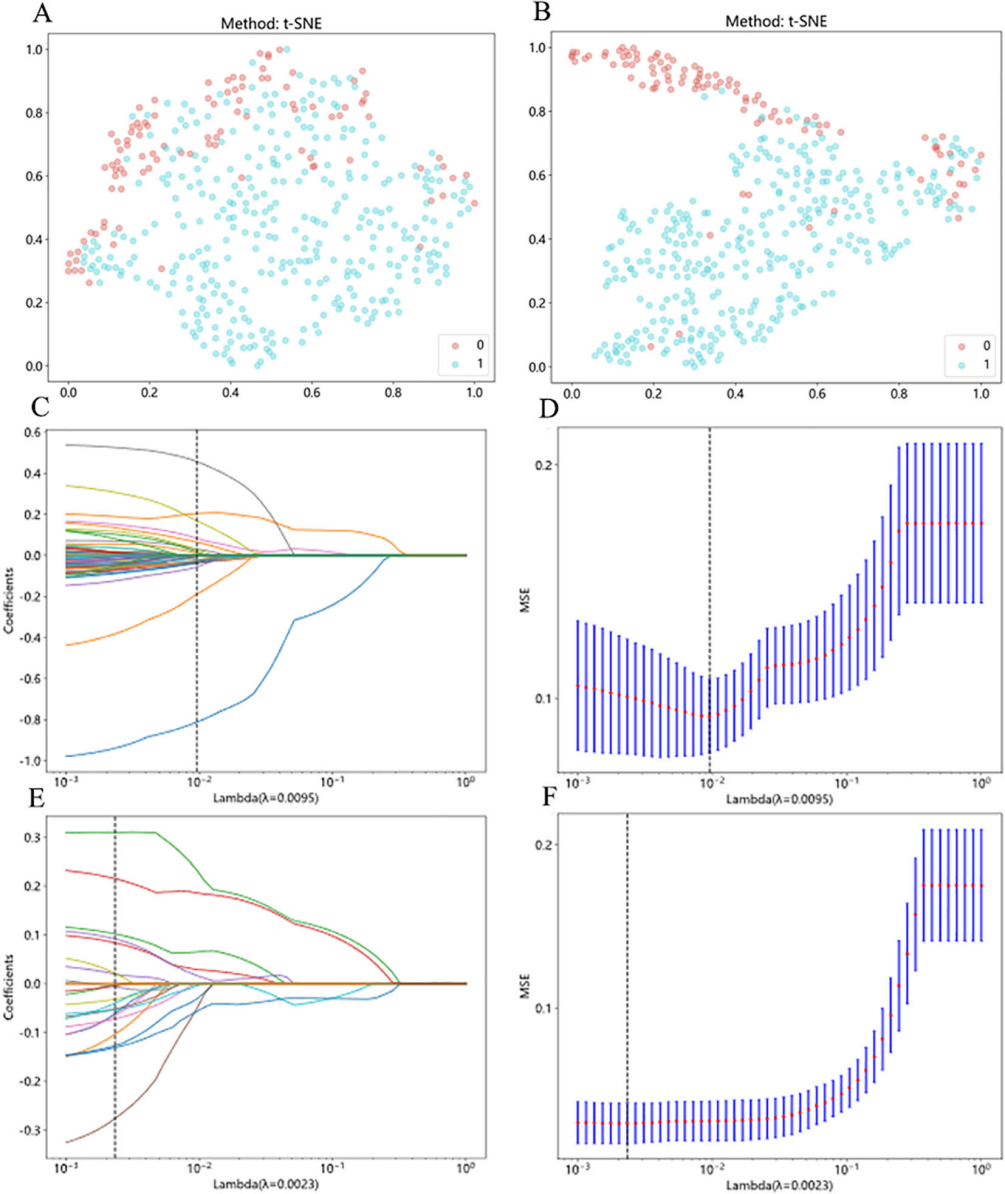
Feature fusion from patch to WSI and LASSO feature selection. **(A)** Sample distribution after t-SNE dimensionality reduction under the supervised strategy. “0” denotes benign samples, and “1” denotes malignant samples. **(B)** Sample distribution after t-SNE dimensionality reduction under the weakly supervised strategy. “0” denotes benign samples, and “1” denotes malignant samples. **(C, D)** LASSO regression process for samples under the supervised strategy. “Coefficients” represent the magnitude of the feature coefficients, and “MSE” refers to Mean Squared Error. **(E, F)** LASSO regression process for samples under the weakly supervised strategy. “Coefficients” represent the magnitude of the feature coefficients, and “MSE” refers to Mean Squared Error.

We performed feature fusion using the PLH and BoW pipelines on features extracted from the supervised and weakly supervised InceptionV3 models to obtain features for WSI pathological images. After applying t-distributed Stochastic Neighbor Embedding (t-SNE) for dimensionality reduction, we found distinct differences between benign/malignant samples, with the weakly supervised strategy showing greater distinction (**Figures 3A, B**). Subsequently, we used a LASSO regression model for feature selection, determining the optimal λ value that minimized the Mean Squared Error (MSE). Features with non-zero coefficients at this λ value were selected as inputs for machine learning (**Figures 3C–F**). The supervised strategy retained 15 features with non-zero coefficients, while the weakly supervised strategy retained 13.

**Figure 4:**
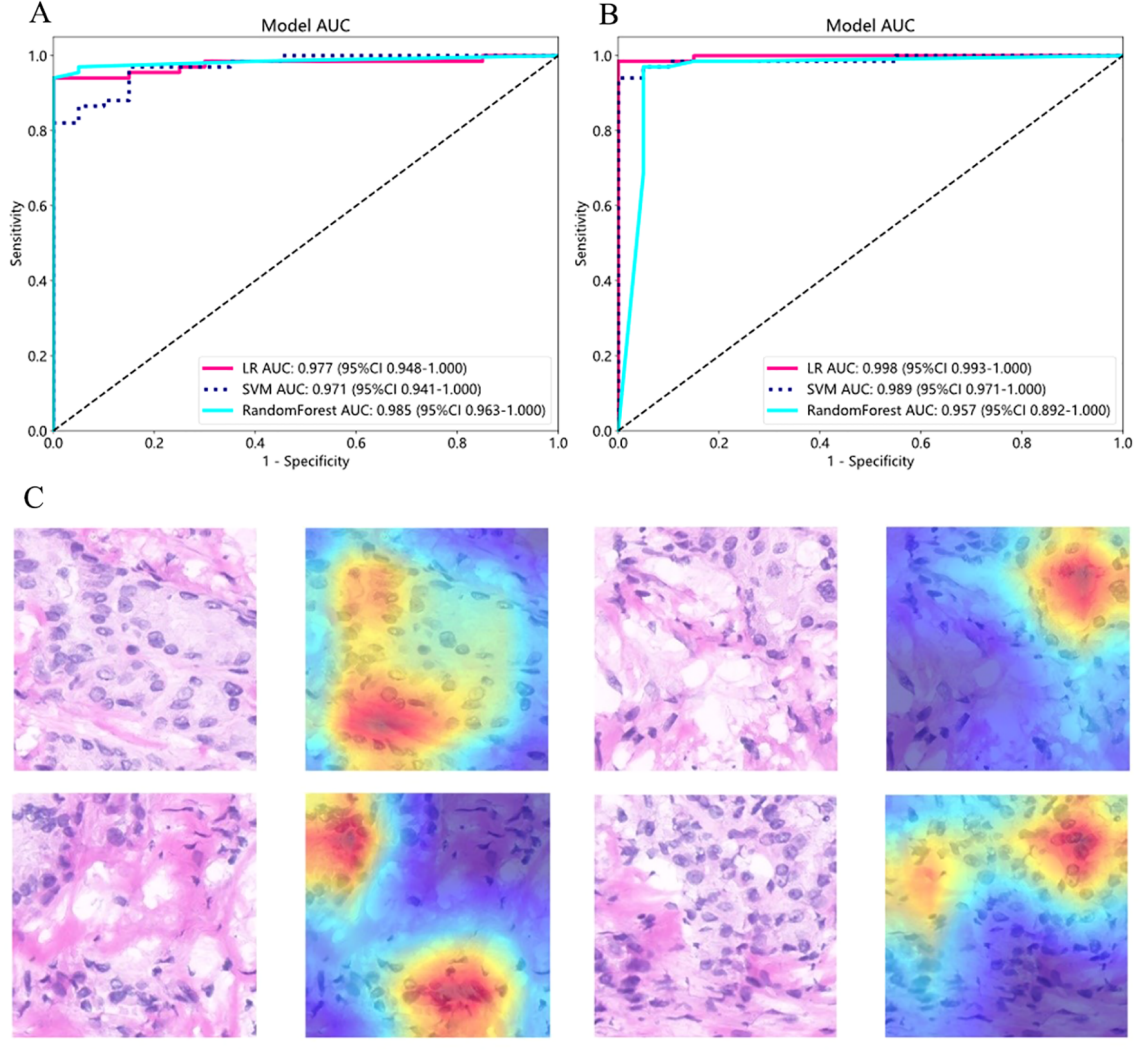
WSI-level prediction results and regional heatmaps. **(A)** ROC curve for WSI-level supervised strategy. **(B)** ROC curve for WSI-level weakly supervised strategy. **(C)** Examples of Heatmaps for Four Regions of Interest. On the left are cropped patches with a size of 512×512 pixels, and on the right are the corresponding heatmaps, where red regions indicate areas attended by the model. LR, Logistic Regression; SVM, Support Vector Machine; AUC, Area Under the Curve.

We then constructed three models—LR, SVM, and RF—for predicting benign/malignant WSIs, evaluating their performance using accuracy and AUC values (**Figures 4A, B**). The RF model under the supervised strategy achieved the highest AUC of 0.985, while the LR model under the weakly supervised strategy performed best with an AUC of 0.998. Both strategies showed significant improvements from patch-level to WSI-level AUC, indicating that feature aggregation via BoW and PLH pipelines enhanced the WSI-level classification model. Notably, although the weakly supervised strategy had a lower patch-level AUC, it outperformed the supervised strategy at the WSI-level, demonstrating a more pronounced improvement in performance.

**Figure 5:**
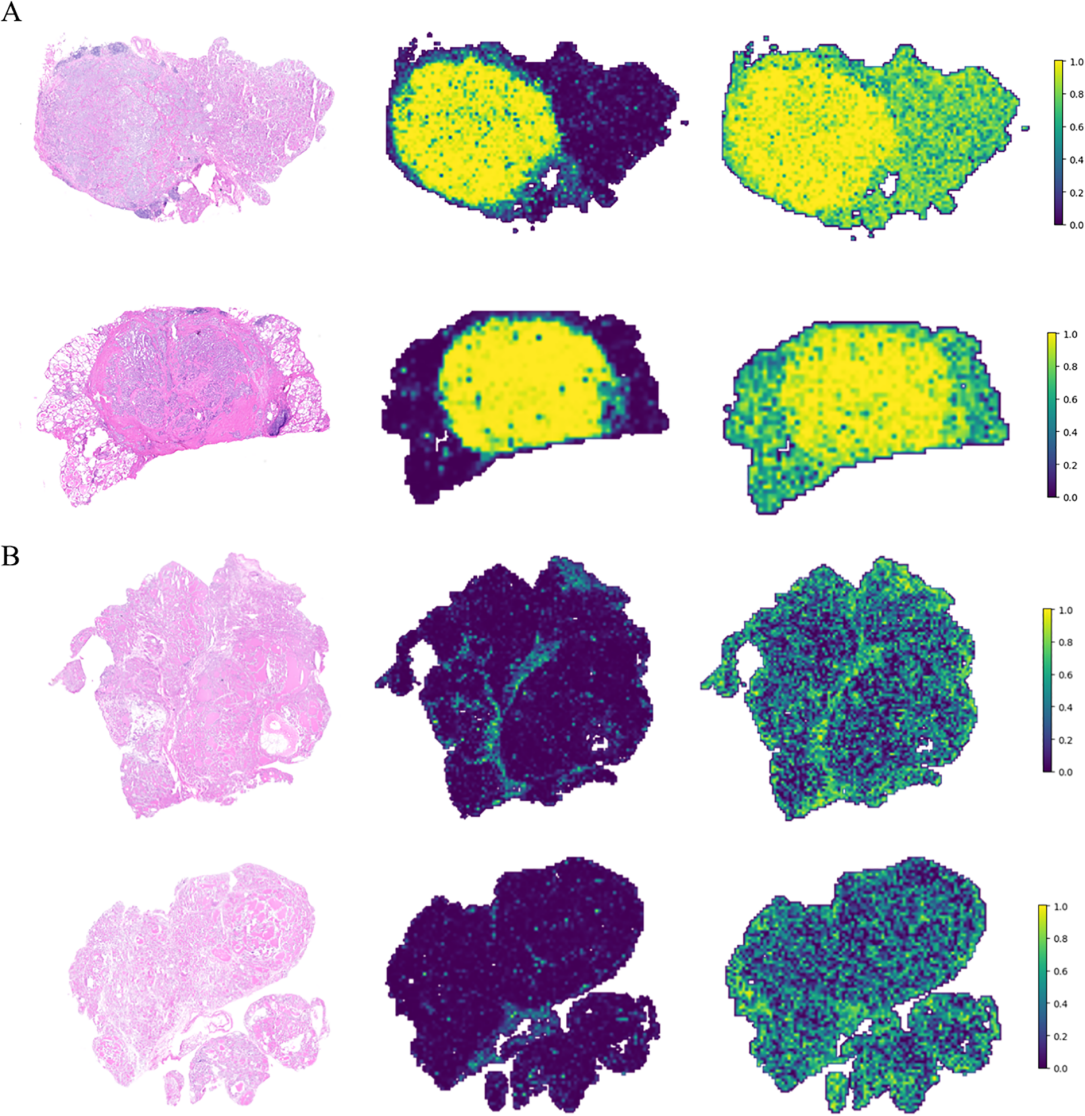
Prediction probability maps of patch classification models. **(A)** Two malignant samples (Left) with prediction probability maps under supervised (Middle) and weakly supervised (Right) strategies. **(B)** Two benign samples (Left) with prediction probability maps under supervised (Middle) and weakly supervised (Right) strategies.

To enhance model interpretability, we applied the Gradient-Weighted Class Activation Mapping (Grad-CAM) algorithm to visualize the activation of the last convolutional layer in the InceptionV3 network. The red regions on the heatmap indicate the areas the model focuses on within the image (**Figure 4C**). These regions primarily correspond to enlarged cell nuclei and areas of nuclear division, which are key features of thyroid cancer. Additionally, we aggregated patch-level prediction probabilities to generate category localization maps for the WSI, providing a clear visualization of regions with higher malignancy in thyroid frozen sections (**Figure 5**). Under both strategies, malignant regions were effectively highlighted, and benign samples were accurately predicted. These results demonstrate that classification models using only WSI-level labels can achieve strong performance, identify malignant tumor regions, and validate the feasibility and effectiveness of the weakly supervised strategy.

### 3.3 Prediction of BRAF^V600E^ gene mutation in thyroid cancer

The BRAFV600E mutation status for all samples was determined using real-time PCR-based mutation detection. Among the 436 collected thyroid frozen digital pathology images, 330 contained BRAF^V600E^ gene mutation information, including 301 mutated cases (label set to “1”) and 29 non-mutated cases (label set to “0”). Due to the imbalance between these two classes, 31 mutated WSIs were randomly selected from the mutated group to combine with the 29 non-mutated WSIs, forming a relatively balanced dataset. The 60 WSIs were then split into training and test sets at a 7:3 ratio, comprising 42 WSIs for training and 18 for testing.

**Figure 6:**
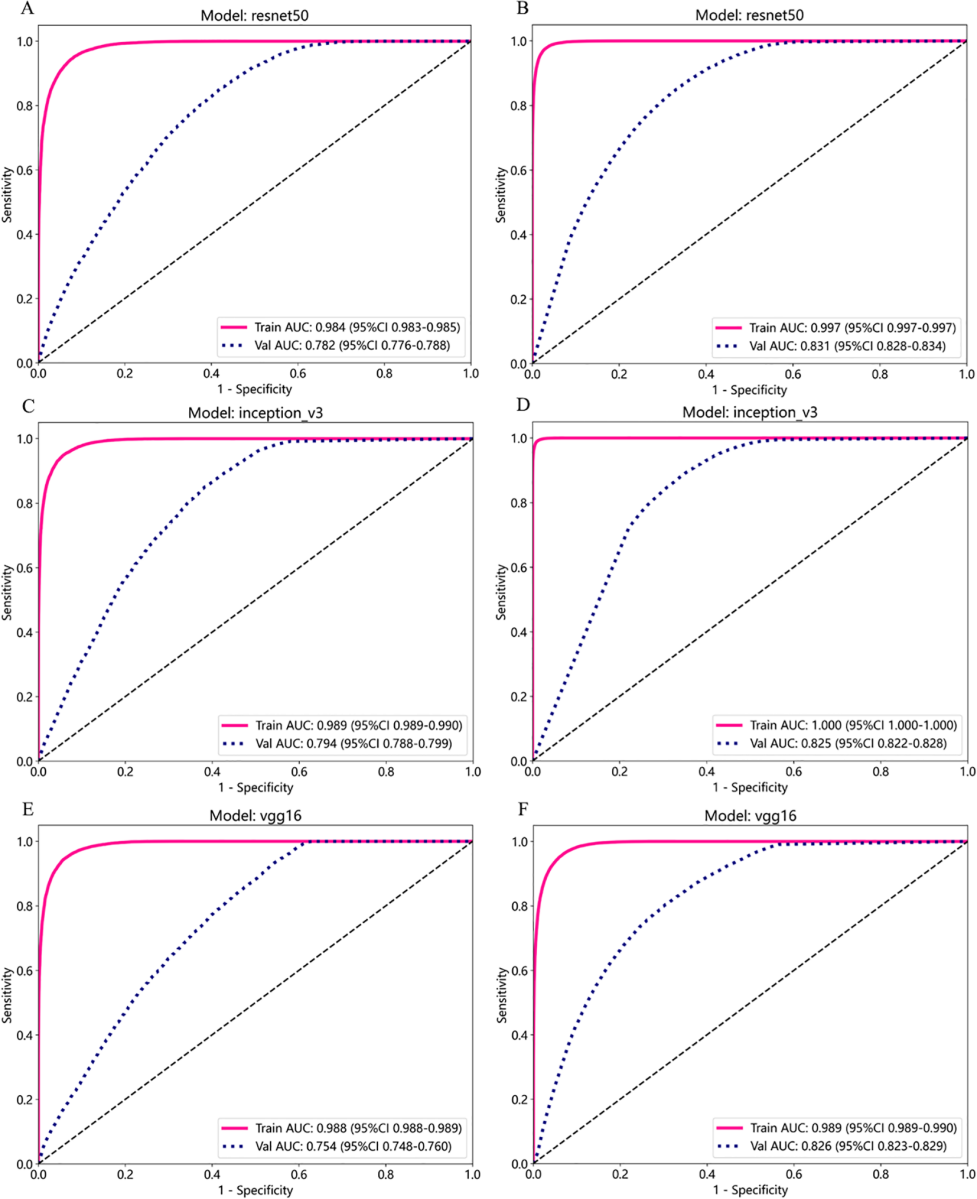
ROC curves of different models in BRAF^V600E^ gene mutation prediction. **(A, B)** ROC curves for the ResNet50 model under conventional **(A)** and extended **(B)** strategies. **(C-D)** ROC curves for the InceptionV3 model under conventional **(C)** and extended **(D)** strategies. **(E, F)** ROC curves for the VGG16 model under conventional **(E)** and extended **(F)** strategies.

The performance metrics of VGG16, InceptionV3, and ResNet50 models under conventional and extended strategies were compared (**Table 2**), and ROC curves were plotted (**Figure 6**). Overall, the performance of the three models under the extended strategy was superior to that under the conventional strategy. Under the conventional strategy, the InceptionV3 model performed the best (AUC = 0.794), while under the extended strategy, the ResNet50 model had the best performance (AUC = 0.831). Therefore, the InceptionV3 model was used as the patch-level classifier under the conventional strategy, while the extended strategy selected the highest-performing ResNet50 model as the classifier for feature extraction to predict WSI classification.

**Table 2:** Performance of different models in BRAF^V600E^ gene mutation prediction.

Training Strategy	Model	AUC	Accuracy	Precision	Sensitivity	F1-score
conventional strategy	ResNet50	0.782	0.671	0.505	0.850	0.633
	InceptionV3	0.794	0.695	0.527	0.849	0.650
	VGG16	0.754	0.615	0.462	0.935	0.619
extended strategy	ResNet50	0.831	0.753	0.692	0.839	0.758
	InceptionV3	0.825	0.762	0.689	0.883	0.774
	VGG16	0.826	0.746	0.695	0.803	0.745

**Figure 7:**
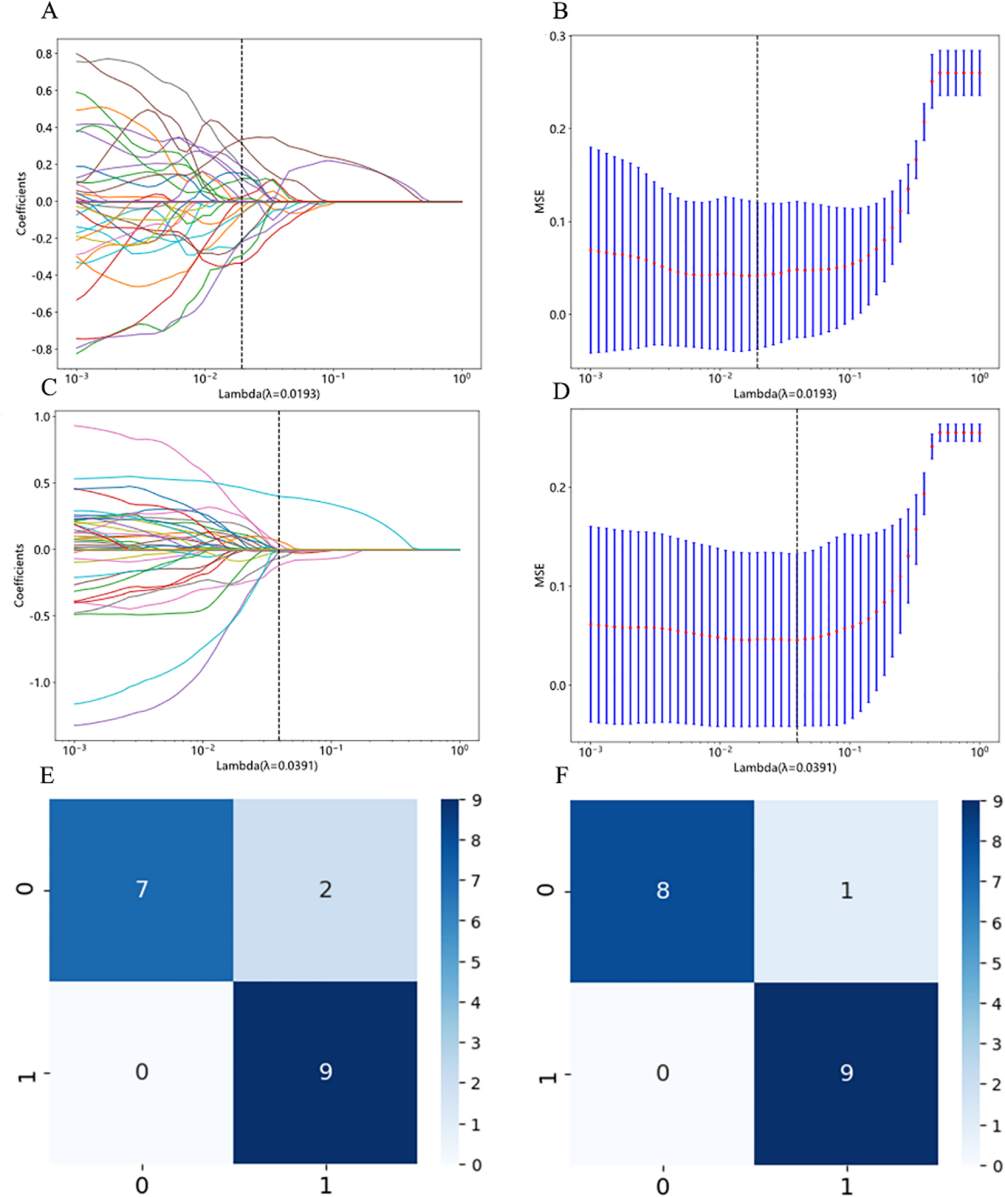
LASSO feature selection and WSI-level prediction results. **(A, B)** LASSO regression process for samples under conventional Strategy. “Coefficients” represent the magnitude of the feature coefficients, and “MSE” refers to Mean Squared Error. **(C, D)** LASSO regression process for samples under extended strategy. “Coefficients” represent the magnitude of the feature coefficients, and “MSE” refers to Mean Squared Error. **(E)** WSI-level prediction results under conventional strategy. On the left, “1” and “0” represent the true BRAF^V600E^ gene mutation and non-mutation, respectively. (F) WSI-level prediction results under extended strategy. On the left, “1” and “0” represent the true BRAF^V600E^ gene mutation and non-mutation, respectively.

Subsequently, we combined features extracted from the conventional InceptionV3 and extended ResNet50 models using both histogram and TF-IDF features. The fused features underwent LASSO regression to eliminate low-correlation or redundant features (**Figures 7A–D**). After LASSO selection, the conventional strategy retained 12 features with non-zero coefficients, while the extended strategy retained 6 features. In the test set of 18 WSIs, the conventional strategy misclassified two mutation-negative WSIs as mutated, whereas the extended strategy had only one false-positive prediction of BRAF^V600E^ mutation (**Figures 7E, F**). This indicates higher prediction accuracy under the extended strategy.

### 3.4 Prediction of lymph node metastasis in thyroid cancer

**Figure 8:**
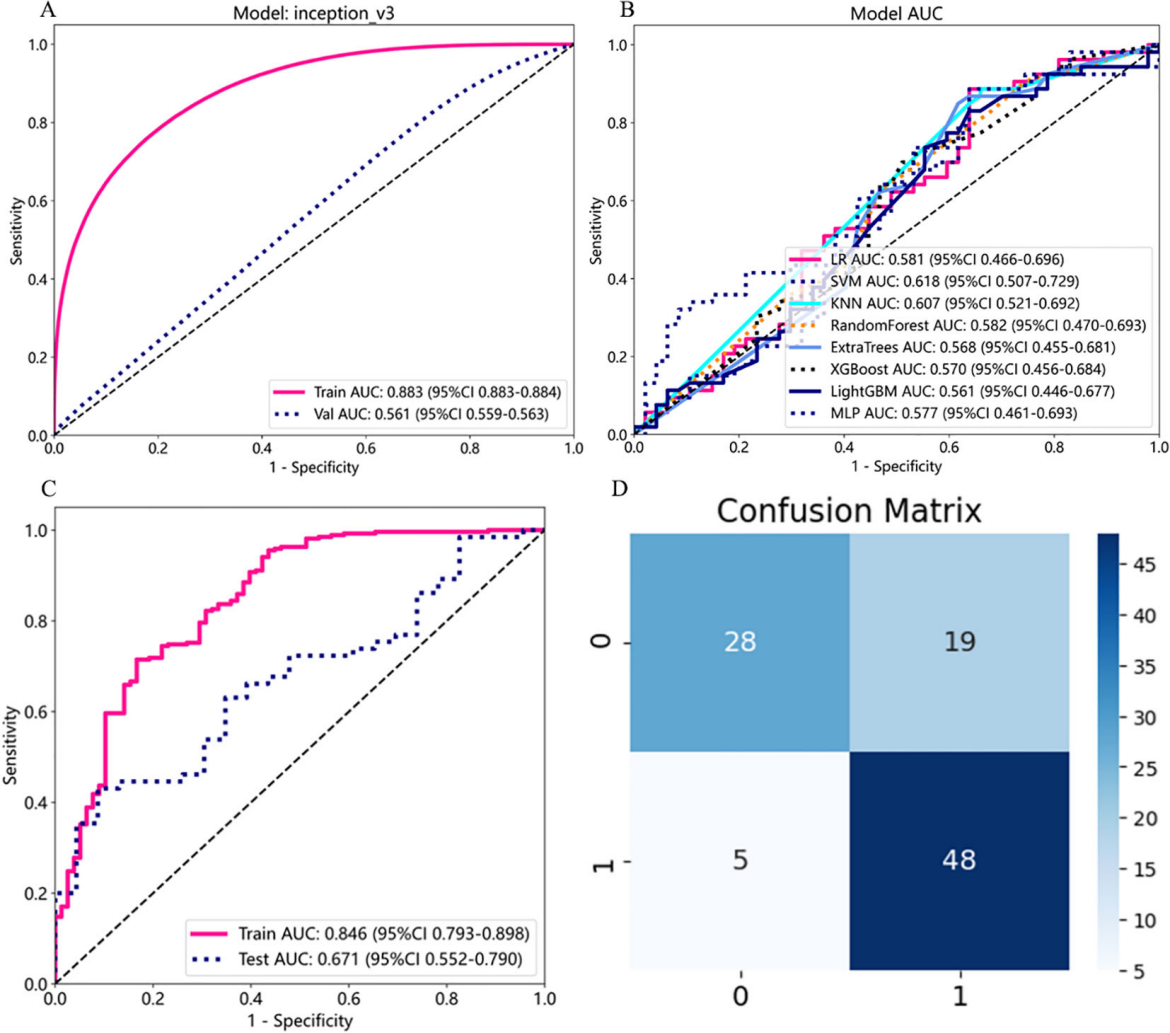
Prediction results for lymph node metastasis in thyroid cancer. **(A)** ROC curve at the patch-level. **(B)** ROC curve at the WSI-level. **(C)** ROC curve at the WSI-level based on the ViT model. **(D)** Confusion matrix for WSI prediction results. On the left, “1” and “0” represent the true presence and absence of lymph node metastasis, respectively.

The InceptionV3 model was used as the patch classifier, and the ROC curve was plotted (**Figure 8A**). The model’s performance was not ideal, with an AUC value of only 0.561 on the test set. Therefore, various machine learning models were used for WSI-level prediction (**Figure 8B**). Although model performance improved slightly, the best-performing SVM among numerous machine learning algorithms only achieved an AUC value of 0.618.

To achieve better performance in predicting lymph node metastasis in thyroid cancer, the ViT network was used as a feature extractor to capture global features, and all patches of the WSI were input to obtain local features. Finally, the ROC curve of the ViT-based prediction model was plotted (**Figure 8C**), with an AUC value of 0.671 on the test set, higher than the AUC value of the CNN model at the WSI-level. Among 100 samples, 24 were incorrectly predicted, with an accuracy rate of 76%, also higher than the CNN model (**Figure 8D**).

### 3.5 Training and validation with TCGA data

We applied the same pipeline to train and validate on thyroid cancer WSI images from the TCGA (The Cancer Genome Atlas) database, comprising 97 normal samples and 200 tumor samples, with a training-to-validation set ratio of 1:1. The deep learning model employed was InceptionV3, and the machine learning models selected were LR, SVM, and RF. The results demonstrated that the RF model achieved an accuracy of 93.3% and an AUC of 0.988, indicating that deep learning methods can effectively classify benign and malignant thyroid WSI samples (**Supplementary Figure S1**).

## 4 Discussion

Thyroid lesions are among the most common specimens requiring intraoperative consultation in current clinical practice. Accurately assessing, classifying, and evaluating the risk of malignancy is the most critical issue in intraoperative diagnosis. However, the sensitivity of frozen sections diagnosis for thyroid nodules is only about 75% (22). Faced with this challenge, AI-assisted diagnosis may be a potential solution. With the development of network architecture and algorithms and the accumulation of medical data, AI has been applied in the diagnosis of thyroid cancer. Since ultrasound examination is the preferred diagnostic tool, AI-assisted diagnosis based on ultrasound images is the most common [For example, the ThyGPT model is a multimodal GPT model for thyroid cancer-assisted diagnosis based on ultrasound images (23)], followed by studies based on cytological pathological images. Studies on AI-assisted intraoperative frozen sections diagnosis are relatively few (24–26), and these studies have all annotated the cancer regions in frozen sections.

We explored whether deep learning can diagnose the benignity/malignancy of thyroid lesions from intraoperative frozen sections with rough cancer region annotation or without cancer region annotation, using only WSI-level labels. At the patch prediction stage, three image block classifiers—VGG16, InceptionV3, and ResNet50—were trained, and the performance of these three CNN networks was comparable. These results validated the effectiveness of selecting the InceptionV3 model for thyroid image block classification through transfer learning. Specifically, for patch-level prediction, models using a supervised learning strategy significantly outperformed those using a weakly supervised strategy, likely because normal regions within cancer samples are prone to misclassification as benign regions under weak supervision.

Currently, studies on benign-malignant classification of WSIs often integrate patch-level into WSI-level predictions based on rules (24), probability heat maps (25), color moments, and voting or probability averaging methods.We adopted another aggregation method, extracting histogram features and TF-IDF feature vectors from patch-level deep learning models and fusing them. We also used three machine learning models—LR, SVM, and RF—all of which achieved good performance, with little difference in results between the two strategies. The results from patch-level AUC to WSI-level AUC in both strategies showed a significant improvement, and the improvement in model performance from patch-level AUC to WSI-level under the weakly supervised strategy was even more pronounced. Comparative experiments showed that in this section’s experimental process, the weakly supervised strategy achieved a level similar to the supervised strategy in the WSI-level classifier. This may be because when malignant tumor regions occupy a very low proportion in malignant samples, WSI-level features under a supervised strategy learn more of the benign region features within malignant tumors, while weakly supervised strategies reduce the possibility of misclassifying malignant samples as benign in such situations. This indicates that the performance of the whole-slide classifier under the weakly supervised strategy is not heavily dependent on the image patch-level classifier, and the success of the weakly supervised strategy proved that good prediction results can be achieved without reliance on pathologists’ annotations.

Studies have shown that BRAF^V600E^ gene mutation detection has high diagnostic value in thyroid nodules with cytological uncertainty and has been proven to be related to the prognosis of thyroid cancer (27). However, recent studies have expressed concerns about the inconsistency between immunohistochemistry detection and the gold standard DNA detection in BRAF^V600E^ gene mutation detection, as the former relies on tissue staining schemes (28, 29). Pathologists traditionally identify morphological features associated with BRAF^V600E^ gene mutation in thyroid cancer through histopathology and cytology (30, 31). However, studies have found that the ability to translate these morphological findings into clinically reliable, effective, and reproducible BRAF^V600E^ gene mutation predictions is limited. Previous studies have also shown this, with the highest observed inter-observer agreement being 0.79, accuracy of 83%, specificity of 71%, and positive predictive value of 78% (30).

Studies have shown that deep learning can extract visual features related to molecular changes from histological images, thereby potentially predicting molecular changes from routine pathological sections (32). Therefore, we used conventional and extended strategies, respectively inputting patches from the ROI and all patches for training. At the patch prediction stage, all models under the extended strategy outperformed those under the conventional strategy, with the ResNet50 model achieving the best AUC of 0.831. The comparison between conventional and extended strategies indicates that information related to BRAF^V600E^ gene mutation in thyroid cancer is not limited to the tumor region of the WSI; pathological features in and around the tumor are also associated with BRAF^V600E^ expression.

Currently, deep learning models based on thyroid pathological images are mainly focused on identifying cancer metastasis areas in lymph node sections. There are few studies predicting LNM based on primary tumor pathological images, and the performance of these models still needs improvement. Therefore, we continued to explore the prediction of lymph node metastasis status from intraoperative frozen sections images of primary thyroid cancer. Initially, we still used the deep learning-machine learning workflow for model training, but the highest AUC value in WSIs was only 0.618. This performance is comparable to that of Wessels (33) and Brinker (34) but falls short of the results achieved by Liu Y et al. (AUC = 0.80 on an internal test set). This may be related to the small sample size from a single institution in this study and the fact that primary tumor pathological images, which were used for lymph node metastasis prediction, contain limited information about lymph node metastasis. Future studies should aim to collect large-scale slide data from multiple centers and incorporate cancer metastasis regions in lymph node slides as well as other clinical information related to lymph node metastasis. Additionally, Liu Y et al. used patches cropped at different magnifications, incorporating images from various magnification levels into the training model. High magnification images better reflect cellular morphology and internal structures, while low magnification images show the overall shape of cells, the distribution pattern of tumor cells, and their surrounding environment. It is evident that histological features at different magnification levels are crucial for LNM prediction.

We used ViT as a feature extractor for model training, aggregating features based on the Transformer framework. Unlike CNN models, the ViT feature extractor can better capture global information. Ultimately, the ViT-based prediction model achieved an AUC value of 0.671 and an accuracy rate of 76% on the test set. Although the performance of this model did not match the excellent performance of the other classification tasks, it demonstrated that deep learning techniques can model the association between lymph node metastasis and related primary tumor histological features. It also indicates that the Transformer framework has certain advantages in LNM prediction tasks, providing insights for future research.

Furthermore, the study has certain limitations. First, existing research on benign-malignant classification using the EfficientNetV2-b0 model enables more nuanced classification of thyroid cancer (35), while studies on lymph node metastasis prediction utilizing ThyNet-LNM have demonstrated robust and excellent predictive performance in multicenter validation (36). Both studies have distinct characteristics that complement this research, collectively forming comprehensive insights into AI-based diagnosis of thyroid cancer frozen sections. This study introduces a weakly supervised strategy for benign-malignant classification and compares the performance of CNN and ViT models for lymph node metastasis prediction. Second, ultrasound is the preferred diagnostic method for thyroid cancer, and frozen section analysis of thyroid tissue has limited clinical application, being used only in specific scenarios (e.g., cases where fine-needle aspiration is diagnosed as “suspicious for malignancy”). Third, the acquisition of WSI images relies on whole-slide digital scanning and computational processing, which may increase time costs and cause potential delays in intraoperative diagnosis. Additionally, the equipment investment is relatively high, which may pose certain challenges for implementation in low-volume medical centers.

## 5 Conclusions

In conclusion, we demonstrate the potential of AI-assisted diagnosis in addressing the challenges of thyroid lesion assessment, particularly in intraoperative frozen sections diagnosis. The use of deep learning models, such as VGG16, InceptionV3, and ResNet50, has shown promise in classifying thyroid lesions with comparable performance. We also highlight the effectiveness of the weakly supervised strategy in achieving results similar to the supervised strategy, reducing reliance on pathologists’ annotations. Furthermore, the exploration of predicting BRAF^V600E^ gene mutation and lymph node metastasis status using deep learning techniques has provided valuable insights, indicating that these models can extract relevant features from histological images. Although the performance of the LNM prediction model was not as high as other classification tasks, it still showed the potential of deep learning in modeling the association between histological features and clinical outcomes. Future research should continue to explore and refine these models, potentially incorporating multi-magnification level images and leveraging the advantages of frameworks like Transformer to improve prediction accuracy and reliability. Additionally, increasing the data volume and diversity could further enhance the robustness and generalizability of these AI models in thyroid cancer diagnosis and prognosis.

## Data Availability

The original contributions presented in the study are included in the article/**Supplementary Material**. Further inquiries can be directed to the corresponding authors.

## Supplementary material

Supplementary Figure 1 Prediction results for the TCGA dataset. **(A–C)** A, B, and C are the confusion matrices for WSI prediction results of the RF, SVM, and LR machine learning models, respectively. The left side shows the sample truth labels. **(D)** ROC curve at the WSI-level based on the machine learning models. RF: Random Forest. SVM: Support Vector Machine. LR: Logistic Regression.

Supplementary Table 1 Final clinical diagnosis and benign/malignant label assignment of samples.
