# Supplementary material for: Deep learning for multitask prediction on thyroid nodule frozen sections
Source: Front Oncol. 2026 Jan 12;15:1676360. doi: 10.3389/fonc.2025.1676360 (PMC12835897; doi:10.3389/fonc.2025.1676360)
Supplement: Supplementary Figure 1 — Prediction results for the TCGA dataset. (A–C) A, B, and C are the confusion matrices for WSI prediction results of the RF, SVM, and LR machine learning models, respectively. The left side shows the sample truth labels. (D) ROC curve at the WSI-level based on the machine learning models. RF: Random Forest. SVM: Support Vector Machine. LR: Logistic Regression. [file Image1.pdf]

Table S1. Final clinical diagnosis and benign/malignant label assignment of samples.

Diagnosis	Count	Label
Nodular Goiter	60	benign
Nodular Goiter with Hashimoto's Disease	13	benign
Hashimoto's Disease	12	benign
Adenomatous Nodular Goiter	5	benign
Thyroid Adenoma	3	benign
Nodular Goiter with Cystic Degeneration	2	benign
Subacute Thyroiditis with Hashimoto's Disease	2	benign
Normal	2	benign
Nodular Goiter with Cystic Degeneration and Squamous Metaplasia	1	benign
Adenomatous Nodular Goiter with Hashimoto's Disease	1	benign
Papillary Thyroid Carcinoma	298	malignant
Papillary Thyroid Carcinoma with Hashimoto's Disease	30	malignant
Medullary Thyroid Carcinoma	2	malignant
Encapsulated Papillary Thyroid Carcinoma	1	malignant
Multiple Papillary Thyroid Carcinoma with Hashimoto's Disease	1	malignant
Papillary Thyroid Carcinoma with Nodular Goiter	1	malignant
Papillary Thyroid Carcinoma with Subacute Thyroiditis	1	malignant
Papillary Thyroid Microcarcinoma with Hashimoto's Disease	1	malignant

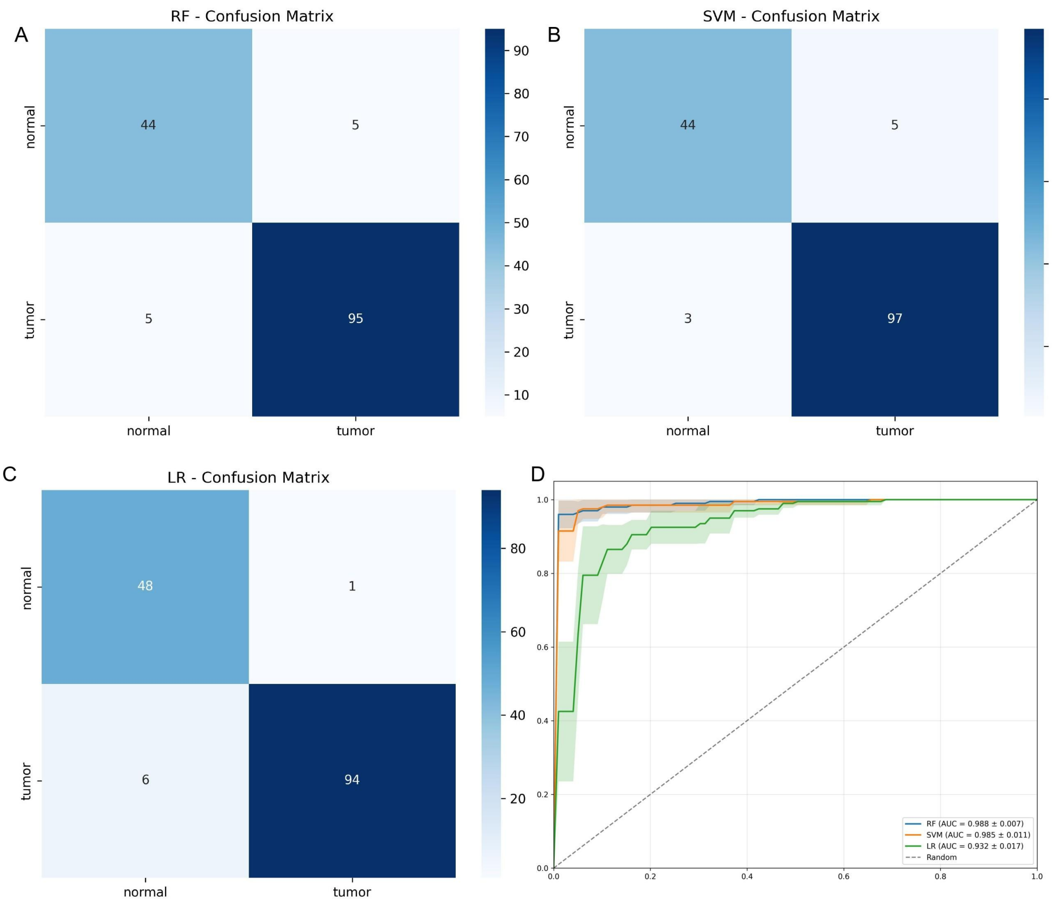

Figure S1. Prediction results for the TCGA dataset. (A – C) A, B, and C are the confusion matrices for WSI prediction results of the RF, SVM, and LR machine learning models, respectively. The left side shows the sample truth labels. (D) ROC curve at the WSI-level based on the machine learning models. RF: Random Forest. SVM: Support Vector Machine. LR: Logistic Regression.
